# Characterization of Malectin/Malectin-like Receptor-like Kinase Family Members in Foxtail Millet (*Setaria italica* L.)

**DOI:** 10.3390/life13061302

**Published:** 2023-05-31

**Authors:** Xiuqing Jing, Ning Deng, Abdullah Shalmani

**Affiliations:** 1 Department of Biology, Taiyuan Normal University, Jinzhong 030619, China; ddd_denny@163.com; 2 College of Life Science, Shanxi University, Taiyuan 030006, China; 3 National Key Laboratory for Crop Genetic Improvement, Huazhong Agricultural University, Wuhan 430070, China; abdullahshalamni@nwafu.edu.cn

**Keywords:** malectin-like receptor-like kinase, transcriptional profile, abiotic stresses, phytohormone, foxtail millet (*Setaria italica*)

## Abstract

Plant malectin/malectin-like receptor-like kinases (MRLKs) play crucial roles throughout the life course of plants. Here, we identified 23 SiMRLK genes from foxtail millet. All the SiMRLK genes were named according to the chromosomal distribution of the *SiMRLKs* in the foxtail millet genome and grouped into five subfamilies based on phylogenetic relationships and structural features. Synteny analysis indicated that gene duplication events may take part in the evolution of SiMRLK genes in foxtail millet. The expression profiles of 23 SiMRLK genes under abiotic stresses and hormonal applications were evaluated through qRT-PCR. The expression of *SiMRLK1*, *SiMRLK3*, *SiMRLK7* and *SiMRLK19* were significantly affected by drought, salt and cold stresses. Exogenous ABA, SA, GA and MeJA also obviously changed the transcription levels of *SiMRLK1*, *SiMRLK3*, *SiMRLK7* and *SiMRLK19*. These results signified that the transcriptional patterns of *SiMRLKs* showed diversity and complexity in response to abiotic stresses and hormonal applications in foxtail millet.

## 1. Introduction

Foxtail millet (*Setaria italica*) is one of the main food crops and is cultivated in arid, semi-arid and barren areas of northern China and India [1]. Foxtail millet is highly adaptable to adverse growing conditions and is planted with fewer inputs, but it has excellent nutritional properties [2]. The seeds of foxtail millet are rich in protein composition and high in essential amino acids, making them one of the most important healthy foods [1,3]. In addition, the germplasm resources of foxtail millet are abundant, with the largest number of cultivated and wild types, which has a good application prospect in crop improvement projects such as gene mapping, allelic gene mining, and selection of excellent varieties [4]. Moreover, the genome sequencing of foxtail millet has been completed [5]. Its genome size is relatively small, approximately 515 Mb, and its life cycle is short, making it an ideal model system for crop research.

Plant cells can sense and respond to external stress signals through pattern recognition receptors (PRRs), including receptor-like proteins (RLP) and receptor-like kinases (RLK) located on the cell surface, so as to make timely responses to stress. RLK plays a key role in the expression of stress-responsive genes by coupling external signals with intracellular signals [6,7,8]. However, the function of the large majority of these RLKs remains to be explored. Among them, there is a subfamily referred to as Malectin/malectin-like domain containing receptor-like protein kinases (MRLKs), also named as the *Catharanthus roseus* RLK1-like (CrRLK1L) protein kinases, which are involved in plant growth, fertilization, hormone signal transduction, immune and stress response [9,10,11,12,13,14,15,16,17]. MRLK proteins feature a predicted intracellular Ser/Thr kinase domain highly conserved among all RLKs, a transmembrane domain and a variable extracellular domain. Within the extracellular domain of MRLK proteins reside one or two malectin/malectin-like modules [11].

Currently, *MRLKs* have been identified in multiple species, and the functions of some of their members have been studied. In *Arabidiposis*, a total of 17 members of the *MRLK* family have been identified, 10 of which have been reported to be involved in plant growth, fertilization, immune response and other aspects [11,13,14,15,16,17]. Up till now, 16 *MRLK* genes have been reported in the rice genome [18], but according to our recent study, at least 67 members were identified in this plant [19]. A total of 31 *MRLK* members that could take part in the response to biotic and abiotic stress have been characterized in the tobacco genome [20,21]; such overexpression of *NtCrRLK1L47* could enhance salt tolerance in tobacco seedlings [20]. A total of *24 CrRLK1L* members were investigated in tomatoes, and they may also be involved in the stress tolerance of tomato [22]. In addition, some studies have shown that *MRLKs* in strawberry are related to fruit ripening and abiotic stress response [23].

In the meantime, the functions of some MRLK homologs have been elucidated. For example, FERONIA (FER), which is the best characterized MRLK family protein, and ANXUR1 (ANX1) and ANXUR2 (ANX2), which are the closest homologues of FER, are key regulators of polar growth and pollen tube reception in the female gametophyte [9,10,11,12,13,24]. OsCrRLK1L2 and OsCrRLK1L3 are involved in the regulation of circadian rhythm [18], OsCrRLK1L15 participates in the response to salt stress [25], and OsMTD2 is essential for pollen tube elongation [26]. PbrCrRLK1L13, in pear, mediates reactive oxygen species signaling and balance of cellulose deposition in pollen tubes [27,28]. However, the biological functions of these MRLKs in foxtail millet are still far from being elucidated.

Therefore, in the current study, based on bioinformatics and qRT-PCR analysis, the *MRLK* family members of millet were identified and characterized, and their possible roles in stress response was studied. A total of 23 members were identified with diverse gene and protein structures and different transcriptional expression levels under various stresses, indicating that they have complex phylogenetic relationships and functional differentiation of the family members to stresses.

## 2. Materials and Methods

### 2.1. Identification of MRLK Family Members in Foxtail Millet

The protein sequences of AtMRLKs that have been identified and characterized [11,16] were used as queries for BLASTP searches in a plant genome database (http://plantgdb.org/SbGDB/SiGDB/BdGDB/) (accessed on 22 October 2022) and the Ensembl Plants website (https://plants.ensembl.org/Setaria_italica/Info/Index) (accessed on 22 October 2022) [29,30]. First, the candidate proteins of foxtail millet were preliminary authenticated by a BLASTP search. Second, we used the protein family database (http://pfam.janel ia.org) (accessed on 6 November 2022) to confirm the MRLK members after finding the malectin (pfam11721) and malectin-like (pfam12819) domain. Then, we browsed through the Ensembl Plants website to obtain the SiMRLK family candidate members. After that, the SMART (http://smart.embl-heidelberg.de/) (accessed on 12 November 2022) [31], Inter Pro Scan program (http://www.ebi.ac.uk/interpro/) (accessed on 12 November 2022) and Conserved Domain Database (CDD) (http://www.ncbi.nlm.nih.gov/cdd/) (accessed on 12 November 2022) [32] were used for further scanning to reaffirm the presence of malectin or malectin-like domains and also to ensure the presence of transmembrane domains and intracellular kinase domains in the candidate proteins. The physicochemical property analysis of MRLK family proteins, such as molecular weight (kD), isoelectric point (pI), aliphatic index, instability index, major amino acids and grand average of hydropathy (GRAVY), were surveyed using the ProtParam (https://www.expasy.org/resources/protparam) (accessed on 2 December 2022) [33].

### 2.2. Distribution of Genes on Chromosomes, Organization of Exons and Introns, and Conserved Amino Acid Motifs Arrangement

The location of the SiMRLK family genes were mapped on the nine chromosomes of millet genomes according to the annotation information in the Ensembl Plants website (https://plants.ensembl.org/Setaria_italica/Info/Index) (accessed on 8 January 2023) [34]. The map was drawn by the Mapchart software (http://www.wageningenur.nl/en.htm) (accessed on 9 January 2023) [35]. Nomenclature of the putative SiMRLK genes was assigned based on their chromosomal order. The organization of exons and introns was constructed using the Gene Structure Display Server (http://gsds.cbi.pku.edu.cn) (accessed on 28 January 2023) [36] by aligning coding sequences (CDS) with their corresponding genomic DNA sequences. The conserved amino acid motifs arrangement were detected using the online version 4.9.1 of the Multiple Expectation for motif elicitation (MEME) tool (http://meme-suite.org/) (accessed on 4 April 2023) with default parameters change to 10 conserved motifs and optimum motif width set to >6 and <200 [36,37].

### 2.3. Phylogenetic Relationship and Syntenic Regions Analysis

To further explore the evolutionary relationship of SiMRLK gene families, the candidate SiMRLKs proteins were initially multiply aligned by using the ClustalW v2.0 online tool (http://www.ebi.ac.uk/Tools/webservices/services/msa/clustalw2_soap) (accessed on 3 April 2023). Then, the neighbor joining phylogenetic tree was constructed by the MEGA 7.0.26 software package [38,39,40] with default parameters and the reliability of interior branches was assessed with 1000 bootstrap repetitions. The syntenic regions occupied by *MRLK* family genes in the foxtail millet genome were explored according to the plant genomic duplication database (http://chibba.agtec.uga.edu/duplication/) (accessed on 26 March 2023). The diagram of syntenic regions analysis was drawn by Circos version 0.63 (http://circos.ca/) (accessed on 26 March 2023) [41].

### 2.4. Plant Material, Growth Conditions, Abiotic Stresses and Hormonal Applications in Foxtail Millet

The foxtail millet accession (jingu 21) was donated by Prof. Han Yuanhuai of Shanxi Agricultural University. In 2022, ‘jingu 21’ was planted in the plant culture room located at the farm at Taiyuan Normal University. The millet plants were grown in seedling trays filled with soil and vermiculite (1:1), alternating between 16 h for 25 °C during the day and 8 h for 20 °C at night, and keeping the relative humidity around 75%. Only healthy and uniform millet plants at the seedling stage (28 days) were selected for the abiotic stresses, which included drought (20% PEG6000), salt (200 mM NaCl) and cold (4 °C), as well as hormonal applications, which included 100 μM abscisic acid (ABA), 100 μM gibberellic acid (GA), 500 μM salicylic acid (SA) and 100 μM methyl jasmonate (MeJA). About 200 millet seedlings were treated with each stress. Three replicates were collected from each stress treatment. Therefore, a total of 600 millet seedlings were used for each stress treatment. The transcriptional level of 6 selected SiMRLKs genes were analyzed at 0, 0.5, 1, 3, 6, 12 and 24 h, respectively. After harvest, the samples were immediately frozen in liquid nitrogen and stored at −80 °C until further analysis.

### 2.5. Total RNA Extraction, cDNA Reverse Transcription, and qRT-PCR Analysis

Total RNA was isolated from millet leaves using TRIzol reagents (Invitrogen, Waltham, MA, USA). The residual genomic DNA was removed by treating the RNA samples with RNase-free DNase. The first strand of cDNA was synthesized from 2 μg of total RNA in 25 µL reaction systems using the M-MLV First Strand Kit (Invitrogen). All the primers for quantitative real-time PCR (qRT-PCR) were designed according to *SiMLRK* sequences using primer 6.0 (Table S3). qRT-PCR was carried out in an Applied Biosystems Quantitative Real-Time PCR Detection System. Each reaction consisted of 10 μL SYBR Premix ExTaq (Takara, Kyoto, Japan), 2 μL cDNA samples, and 1 μL of each primer (10 μM) and 6 μL ddH_2_O in a reaction system of 20 μL. The thermal cycle was as follows: 95 °C for 3 min, followed by 40 cycles at 94 °C for 15 s, 62 °C for 20 s, and 72 °C for 20 s. Melting-curve analysis was performed directly after real-time PCR to verify the presence of gene-specific PCR products. This analysis was done by 94 °C for 15 s, followed by a constant increase from 60 to 95 °C at a 2% ramp rate. The millet actin gene (SiActin1, Transcript ID: Si026509m) was used as an internal control and served as a standard gene for normalizing all mRNA transcriptional levels. The relative amount of template present in each PCR amplification mixture was evaluated by using the 2^−ΔΔCt^ method.

### 2.6. Statistical Analysis

Analysis of variance was performed on the data. The mean and standard deviation of the three replicates for all treatments were compared using the SPSS 11.5 software package (SPSS, Chicago, IL, USA), using the minimum significance difference (LSD) test at the 5% level. Graphics were drawn using Origin 7.5. 

## 3. Results

### 3.1. Identification of SiMRLK Family Members in the Foxtail Millet Genome

**Figure 1:**
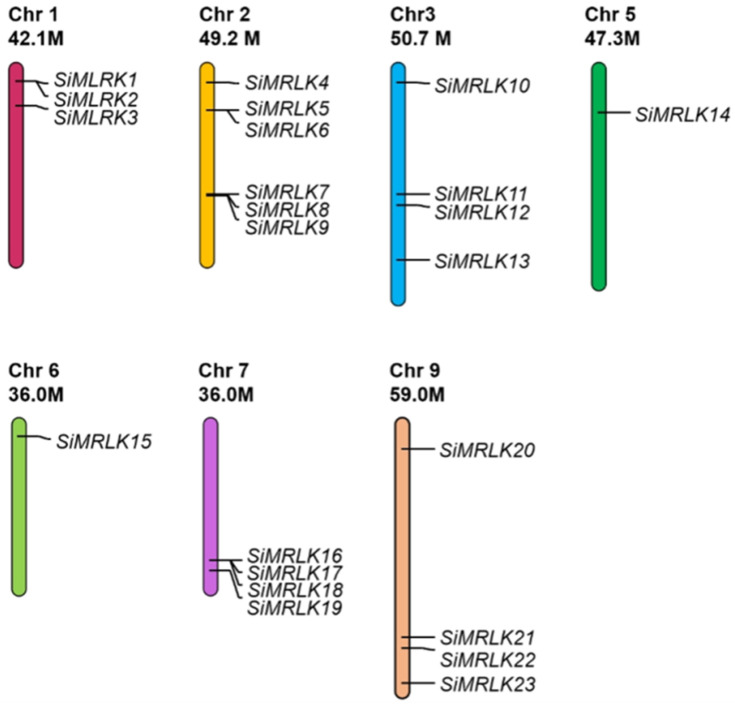
The distribution of 23 SiMRLK genes on chromosomes. Mapchart software was used to map the location of genes on chromosomes. The *SiMRLKs* were distributed on seven chromosomes of millet. The scale is measured in megabases (Mb).

A total of 23 SiMRLK members were identified in the foxtail millet genome (Table 1). The SiMRLK genes were mapped on the chromosomes according to the location information on chromosomes in the foxtail millet genome (Table 1 and Figure 1). We noted that the 23 *SiMRLKs* are distributed on seven of all nine chromosomes in the foxtail millet genome (Figure 1). The majority of *SiMRLKs* (six genes) were mapped on chromosome 2, while one *SiMRLK* was found on chromosome 5 and one on chromosome 6. Furthermore, there are four *SiMRLKs* on chromosomes 3, 7 and 9, respectively, while there are three *SiMRLKs* on chromosome 1 and no SiMRLK gene on chromosomes 4 and 8 (Figure 1). Members of *SiMRLK* family were named *SiMRLK1* to *SiMRLK23* based on their location information on the millet chromosome.

The physiochemical characteristics were studied through the PROTOPARAM online tool (Table S1). The results found that there are many similar characteristics among the members of this family. The assumed lengths of SiMRLK proteins range from 834 (SiMRLK4) to 1082 (SiMRLK5) amino acid residues (Table 1). The molecular masses of the proteins range from 92.17 kD (SiMRLK19) to 118.42 kD (SiMRLK5) (Table S1). All the SiMRLK proteins were found to be hydrophilic according to their grand average of hydropathicity (GRAVY) value. The majority of SiMRLK proteins were acidic in nature according to their isoelectric point (pI), but the pIs of eight SiMRLKs were higher than seven, displaying that the eight SiMRLKs are alkaline proteins in nature (Table S1). Furthermore, the aliphatic index (AI) values range from 79.88 (SiMRLK21) to 94.43 (SiMRLK2). The present study divided 19 SiMLRK members into stable proteins because the instability index of these proteins is less than 40, while the instability index of four proteins (SiMLRK2, SiMLRK13, SiMLRK22 and SiMLRK23) is greater than 40, suggesting that these SiMLRKs are unstable proteins (Table S1). Orientation analysis showed that 10 SiMLRK proteins were located on the forward strand, and the remaining 13 SiMLRK proteins were found on the reverse strand (Table 1). The major amino acid composition of the SiMLRK proteins is Leu, followed by Ser. Some other amino acids, such as Gly, Ala, Thr and Val, are also abundant, varying depending on the different SiMLRK protein (Table S1).

### 3.2. Phylogenetic Analysis and Conserved Motif Analysis of the SiMRLKs in Foxtail Millet

**Figure 2:**
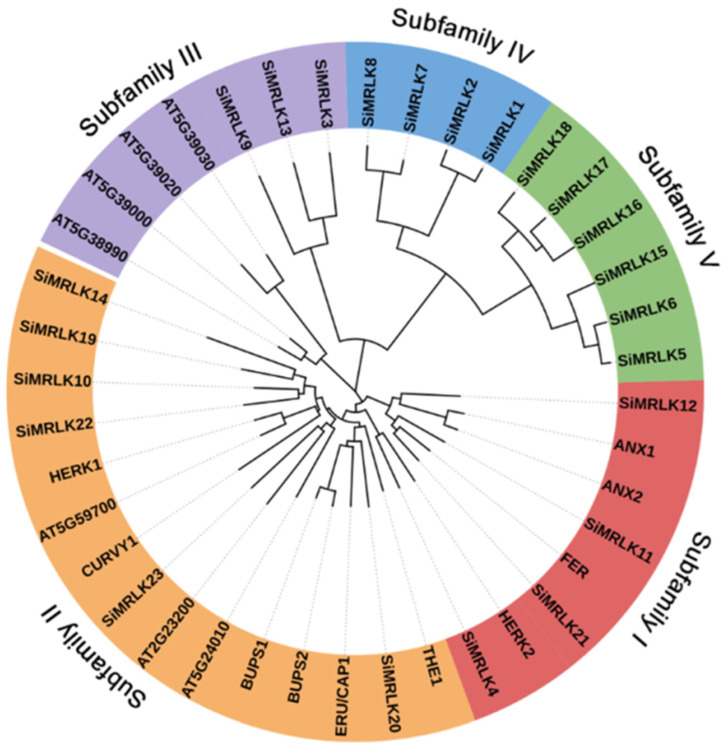
Phylogenetic analysis of MRLKs from *Arabidopsis thaliana* and foxtail millet. Based on the protein sequences of MRLKs from *Arabidopsis* and foxtail millet, the phylogenetic tree was constructed using the method of adjacent linkage (NJ). Subfamilies I–V represent the classification of members of the MRLK family.

In *Arabidopsis*, most of the 17 AtMRLKs have been studied for biological functions. In order to study the phylogenetic relationships of SiMRLKs in millet and predict the possible biological functions of SiMRLKs, we analyzed the phylogenetic relationships of 23 identified SiMRLKs and 17 AtMRLKs (Figure 2). The results showed that the 40 MRLK proteins in the phylogenetic tree are divided into five subfamilies (Figure 2). For SiMRLKs, there are four members in group I (SiMRLK4, −11, −12, −21), six members in subfamily II (SiMRLK10, −14, −19, −20, −22, −23), three members in group III (SiMRLK3, −9, −13), four members in group IV (SiMRLK1, −2, −7, −8), and six members in group V (SiMRLK5, −6, −15, −16, −17, −18). Some SiMRLK proteins are tightly grouped with the AtMRLK proteins (such as group I, II and III), indicating that these proteins are evolutionarily closely related to AtMRLKs and that they may perform similar biological functions in different species (Figure 2).

**Figure 3:**
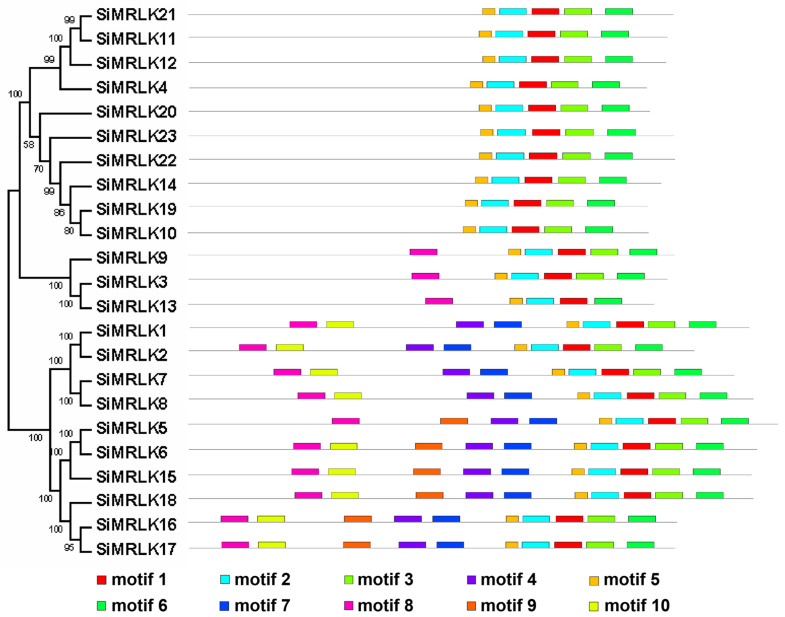
The motif distributions and three-dimensional structures of SiMRLK proteins. The motifs were numbered 1–10, with different colored boxes representing different motifs. The sequence information of each motif is shown in Table S2.

To further study the architecture of the SiMRLK family members, we identified the conserved amino acid motif arrangement of 23 identified SiMRLK proteins. Ten motifs, named as motifs 1 to 10, were detected by MEME analysis (Figure 3 and Table S2). Based on the analysis results, it can be found that motifs 1, 2, 3, 5, and 6 are widely distributed among all family members and are key motifs of the SiMRLK family proteins in foxtail millet. Meanwhile, similar motif composition and assembly order are conserved among members of the same subfamily of SiMRLK (Figure 3). For instance, the subfamily I and II just contained motifs 5-2-1-3-6 in order, and the subfamily III contained motifs 8-5-2-1-3-6 in order, while 8-10-4-7-5-2-1-3-6 in order appeared in subfamily IV and 8-10-9-4-7-5-2-1-3-6 in subfamily V. However, there is a special motif, motif 9, found only in the proteins of the V subfamily (Figure 3 and Table S2).

### 3.3. Structural Diversity and Duplication Analysis of SiMRLK Genes in Foxtail Millet

In order to clarify the intron–exon organization of SiMRLK family genes, a gene structure diagram was constructed based on the CDS sequence and genomic DNA sequence of each member of the SiMRLK family, which can clearly display the distribution position of each exon and intron in its own gene.

**Figure 4:**
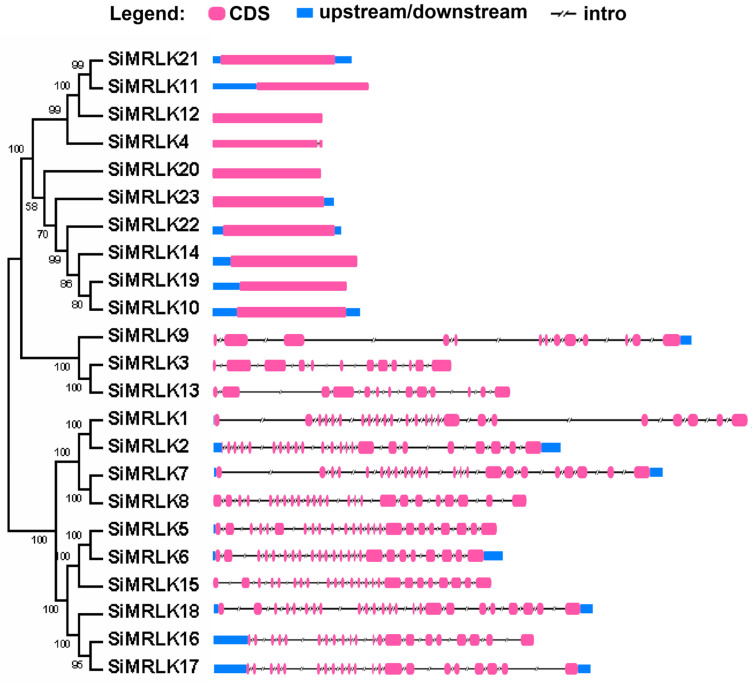
Exon-intron compositions of the SiMRLK family members. Names of genes are indicated on the left. The exon–intron composition of each *SiMRLKs* were showed on the right-hand side.

We found that intron–exon organization and distribution is different among the 23 SiMRLK genes (Figure 4), but the genes in each subfamily usually have similar exon–intron organization and distribution. For example, all the genes in subfamilies I and II are intron-free except for *SiMRLK4*, which contains only one intron. Similarly, we note that the genes in subfamily III have 12 or 13 introns. In addition, the intron–exon organization and distribution of subfamily IV and V genes is very similar, with 20 to 23 introns (Figure 4).

**Figure 5:**
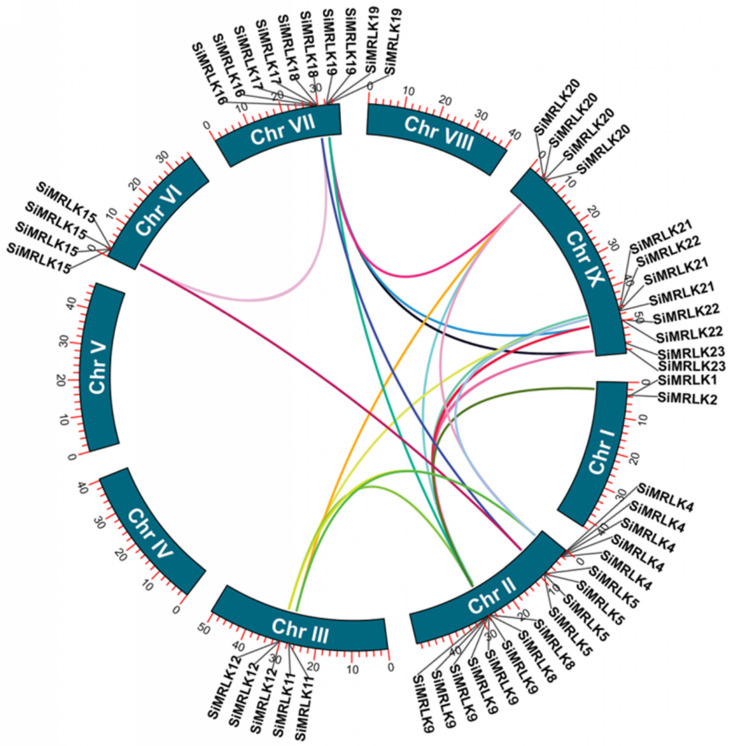
Synteny analysis of *SiMRLKs* in foxtail millet. Nine chromosomes of millet are shown in the circle. The position of *SiMRLKs* on the chromosome were marked on the circle. The curve displayed the duplication relationship between genes in the SiMRLK family.

The duplication of a single gene, a segment of a chromosome or the entire genome itself is crucial to the evolution of a gene family in biology, because the emergence of new genes and their new biological functions depends on these genes or chromosome segments generated by duplication [42]. We tested the duplicated regions that were present in all members of the SiMRLK family to verify if there were the events of genes or chromosome fragments duplication that were present during the evolution of the family. The results revealed that 19 *SiMRLK* pairs of the duplicated region exist in the SiMRLK family genes (Figure 5), signifying that evolutionary events may take part in the evolution of SiMRLK genes in foxtail millet.

### 3.4. Transcriptional Profiles of SiMRLK Family Genes under Abiotic Stresses and Phytohormone

**Figure 6:**
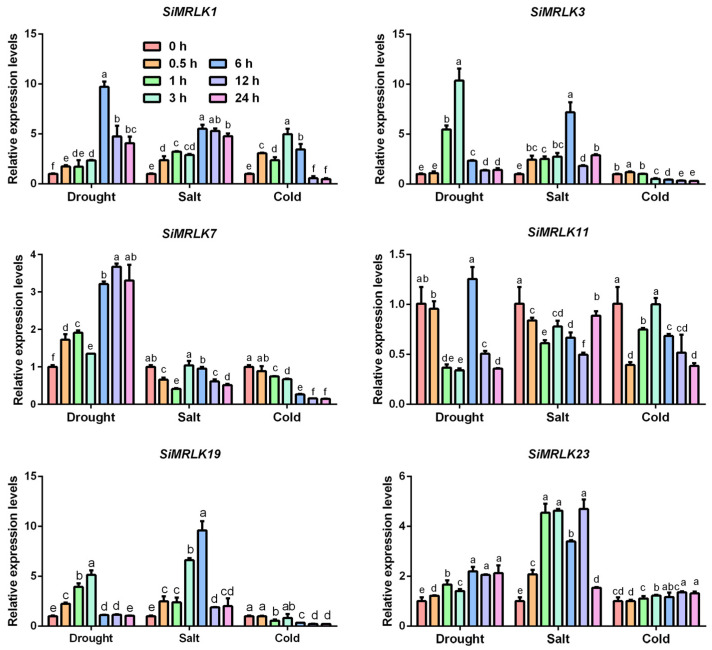
Transcriptional expression profiles of the selected six *SiMRLKs* in responding to abiotic stresses. Four-week-old millet seedlings were used for abiotic stress. All of the experiments were repeated three times, with lowercase letters (a–f) representing significant differences (*p* < 0.05).

To explore whether *SiMRLKs* are involved in the response to abiotic stresses, qRT-PCR were carried out to investigate the transcript levels of SiMRLK gene family members. It is exhaustively difficult to describe the expression profiles of all 23 SiMRLK genes; therefore, six SiMRLK members of the SiMRLK gene family (*SiMRLK1*, *SiMRLK3*, *SiMRLK7*, *SiMRLK11*, *SiMRLK19* and *SiMRLK23*) were assessed. As shown in Figure 6, under drought stress, the transcript levels of *SiMRLK1*, *SiMRLK3*, *SiMRLK7* and *SiMRLK19* were up-regulated, whereas that of *SiMRLK11* was down-regulated. The expression levels of *SiMRLK1*, *SiMRLK19* and *SiMRLK23* were higher than 0 h (control) at most of the test points, while the transcriptional levels of *SiMRLK7* and *SiMRLK11* were down-regulated at most test points under salt stress. The expression of *SiMRLK7* was significantly up-regulated at 9 h and slightly up-regulated at the remainder of the time points. Moreover, some *SiMRLK* members (*SiMRLK7* and *SiMRLK19*) were down-regulated at all tested time points, while the other four *SiMRLKs* showed different transcription levels at different time points under cold stress. The results indicate that although the expression patterns of genes vary under different stresses, the transcription levels of all tested SiMRLK genes undergo significant changes after being subjected to drought, salt, and cold stresses. The difference is that the transcription levels of most genes are up-regulated after being subjected to drought and salt stress, while the transcription levels decrease after being subjected to cold stress.

**Figure 7:**
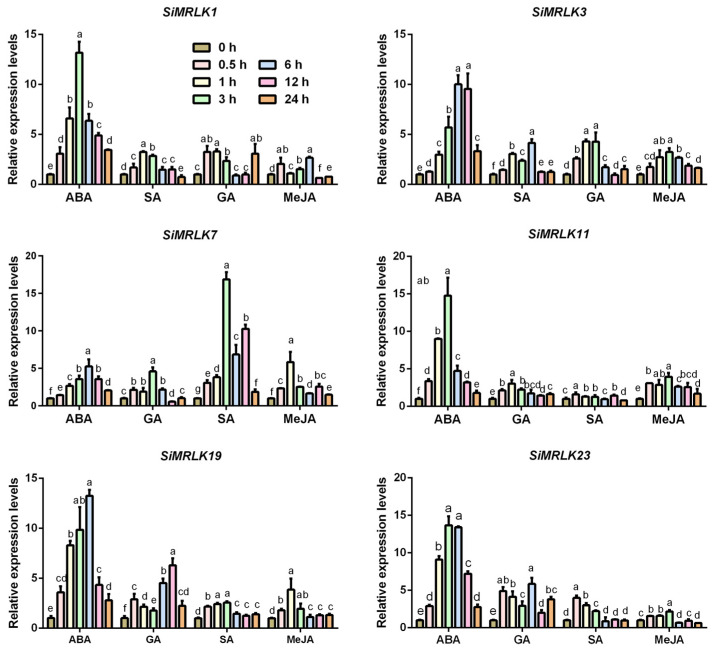
Transcriptional expression profiles of the selected six *SiMRLKs* in responding to hormonal applications. Four-week-old millet seedlings were treated by the hormones. All of the experiments were repeated three times, with lowercase letters (a–f) representing significant differences (*p* < 0.05).

We also tested the transcription levels of six tested *SiMRLKs* after exogenous application of phytohormone ABA, SA, GA, and MeJA (Figure 7). The expression levels of all tested genes are significantly up-regulated at the early time points after exogenous application of phytohormone ABA and then decreased. Application of phytohormone SA significantly up-regulated the expression levels of all tested SiMRLK genes at the most points, except *SiMRLK1* at 24 h and *SiMRLK7* at 12 h. In addition, the transcript level of *SiMRLK7* was strongly stimulated by exogenous GA, while the expression changes of other genes, although up-regulated, were relatively weak. Moreover, exogenous MeJA applications also affected the transcriptional level; especially *SiMRLK3* and *SiMRLK11* were up-regulated at all the tested points. These results indicate that members of the SiMRLK family may be involved in hormone signaling pathways.

## 4. Discussion

MRLKs, also named as CrRLK1L kinases, are widely present in organisms and have multiple important biological functions [16,23,43,44,45]. In plants, it was further reported that MRLK proteins plays an important role in plant growth [46,47], cell wall integrity [16], fertilization [48], hormone signal transduction [14,17,49], and immune and stress response [16,24,45]. The *MRLK* genes have been identified in the genomes of *Arabidopsis* [47], rice [18,19,47], cotton [50], tobacco [21], soybean [51], pear [52] and apple [53]. So far, however, there have been no reports on the members of this family in foxtail millet. In the present study, a total of 23 SiMRLK genes, which are distributed on seven of all nine chromosomes in the foxtail millet genome, were identified through a genome-wide analysis (Figure 1 and Table 1).

We analyzed the phylogenetic relationships of 23 SiMRLKs and 17 AtMRLKs (Figure 2). The results showed that the 40 MRLKs can be divided into five groups according to the results of phylogenetic relationships. Members of group I, II and III were composed of AtMRLKs and SiMRLKs, indicating these SiMRLKs may be orthologous to AtMRLKs and have similar biological functions (Figure 2). Meanwhile, the Group IV and V members contain only SiMRLK proteins, suggesting that these SiMRLKs are slightly more distantly related to AtMRLKs and may have some novel biological functions in addition to those of AtMRLKs.

By analyzing the gene and protein structure of millet SiMRLK family members, it was found that members of each subfamily have similar structures. First, the distribution of motifs among members of the same subfamily is also conservative (Figure 3). For instance, motifs 1, 2, 3, 5, and 6 are widely present in all family members and are arranged in the order of motifs 5-2-1-3-6 from the N-terminal to the C-terminal, which are the motifs that only subfamilies I and II contain and are arranged in this order. Motifs 8-5-2-1-3-6 in order appeared in subfamily III. Meanwhile, motifs 8-10-4-7-5-2-1-3-6 existed in subfamily IV, and motifs 8-10-9-4-7-5-2-1-3-6 arrangement in subfamily V (Figure 3 and Table S2). Second, the distribution and number of intron–exons are conserved among the members of the same subfamily. For example, all genes in subfamily I and II, excluding *SiMRLK4*, possess intronless.There are 12 or 13 introns in the genes of Subfamily III, and 20–23 introns in the subfamily IV and V genes (Figure 4). Third, 19 duplicated pairs are detected in the SiMRLK gene family in foxtail millet (Figure 5). These results suggested that gene duplication events may play an important role in the functional diversification of SiMRLK family genes in foxtail millet. Overall, members of the same subfamily of the SiMRLK family have similar intron–exon distribution and conserved motif arrangement, and duplication events also exist among the genes in this family, revealing a close evolutionary relationship among members of the SiMRLK family in foxtail millet.

Research has discovered that FER positively participates in and regulates auxin and brassinosteroid responses [54,55], facilitates the cross-talk between hormones and RALF peptides in cell growth and stress responses [56,57], and integrates with hormone signaling to regulate plant growth, immune and stress responses [58,59,60,61,62]. Here, we noted that the transcription levels of the majority of tested SiMRLK family genes are significantly affected by abiotic stress and plant hormones, suggesting that these *SiMRLKs* may play a key role in response to abiotic stress in plants, and also participate in hormone signaling pathways. However, the functional mechanism of the SiMRLK family in millet and its possible biological contribution need further molecular physiological experiments.

## 5. Conclusions

In this study, 23 SiMRLK members were identified and renamed according to the chromosomal distribution and grouped into five subfamilies based on phylogenetic relationships. By analyzing the structural characteristics of SiMRLK family members, we found that the members of each subfamily possess a similar structure, such as similar motif composition and conserved intron–exon distribution. Synteny analysis suggests that gene duplication events may be involved in the diversification process of *SiMRLKs* function in foxtail millet. The expression profiles of the SiMRLK genes, evaluated through qRT-PCR, suggest that the SiMRLK genes are responsive to a number of plant hormones and may play a key role in responding to multivariable abiotic stress. These results indicate that members of the SiMRLK family may play an important role in plant responses to abiotic stresses and hormone signal transduction.

## Figures and Tables

**Table 1:** Identification of *MRLK* family genes in in foxtail millet genome.

Name	Gene	Genomic Location	Orientation	DNA	mRNA	PROTEIN	Exons
*SiMRLK1*	SETIT_016192mg	Chr I: 2,269,336-2,281,707	Reverse	12372	3075	1020	24
*SiMRLK2*	SETIT_016251mg	Chr I: 2,286,255-2,294,201	Reverse	7947	3395	922	23
*SiMRLK3*	SETIT_016287mg	Chr I: 7,753,767-7,759,223	Forward	5457	2622	873	12
*SiMRLK4*	SETIT_028931mg	Chr II: 2,466,757-2,469,304	Reverse	2548	2505	834	2
*SiMRLK5*	SETIT_028769mg	Chr II: 8,797,129-8,803,723	Reverse	6595	3290	1082	24
*SiMRLK6*	SETIT_028790mg	Chr II: 8,808,248-8,814,896	Reverse	6649	3602	1036	24
*SiMRLK7*	SETIT_028810mg	Chr II: 27,658,950-27,669,289	Forward	10340	3325	994	23
*SiMRLK8*	SETIT_028799mg	Chr II: 27,683,557-27,690,810	Forward	7254	3090	1029	24
*SiMRLK9*	SETIT_028878mg	Chr II: 27,824,611-27,835,635	Forward	11049	2916	887	13
*SiMRLK10*	SETIT_021217mg	Chr III: 2,531,201-2,534,600	Forward	3400	3400	840	1
*SiMRLK11*	SETIT_021180mg	Chr III: 27,523,701-27,527,334	Forward	3634	3634	873	1
*SiMRLK12*	SETIT_024804mg	Chr III: 29,987,665-29,990,274	Reverse	2610	2610	869	1
*SiMRLK13*	SETIT_024630mg	Chr III: 42,311,418-42,318,343	Forward	6926	2547	848	13
*SiMRLK14*	SETIT_000277mg	Chr V: 9,293,577-9,296,524	Forward	2948	2948	862	1
*SiMRLK15*	SETIT_013178mg	Chr VI: 2,184,449-2,190,852	Reverse	6404	3084	1027	24
*SiMRLK16*	SETIT_009322mg	Chr VII: 29,953,522-29,960,949	Reverse	7428	3471	891	21
*SiMRLK17*	SETIT_009325mg	Chr VII: 29,970,651-29,979,365	Reverse	8715	3703	886	21
*SiMRLK18*	SETIT_009240mg	Chr VII: 30,011,401-30,020,155	Reverse	8755	3481	1029	24
*SiMRLK19*	SETIT_009354mg	Chr VII: 32,306,664-32,309,798	Forward	3135	3135	836	1
*SiMRLK20*	SETIT_039238mg	Chr IX: 5,036,546-5,039,071	Forward	2526	2526	841	1
*SiMRLK21*	SETIT_034180mg	Chr IX: 47,183,943-47,187,152	Reverse	3210	3210	884	1
*SiMRLK22*	SETIT_034215mg	Chr IX: 49,665,610-49,668,585	Reverse	2976	2976	863	1
*SiMRLK23*	SETIT_034221mg	Chr IX: 57,483,937-57,486,745	Reverse	2809	2809	861	1

## Data Availability

All data supporting the findings of this study are available within the paper and its supplementary materials published online.

## Supplementary Materials

The following supporting information can be downloaded at: https://www.mdpi.com/article/10.3390/life13061302/s1; Table S1. Analysis of physicochemical properties of SiMRLK family proteins. Table S2. The conserved motif analysis of the SiMRLK family proteins in foxtail millet. Table S3. Primer sequences for qRT-PCR used in the study.
