# Supplementary material for: Characterization of Malectin/Malectin-like Receptor-like Kinase Family Members in Foxtail Millet (Setaria italica L.)
Source: Life (Basel). 2023 May 31;13(6):1302. doi: 10.3390/life13061302 (PMC10303277; doi:10.3390/life13061302)
Supplement: Supplementary file 1 [file life-13-01302-s001.zip › life-2401191-supplementary.pdf]

Supplemental materials

Characterization of Malectin/Malectin-like Receptor-like Kinase Family Members in Foxtail Millet (*Setaria italica* L.)

Xiuqing Jing [1,2,*], Ning Deng [1] and Abdullah Shalmani [3]

1 Department of Biology, Taiyuan Normal University, Jinzhong 030619, China; ddd_denny@163.com
2 College of Life Science, Shanxi University, Taiyuan 030006, China
3 National Key Laboratory for Crop Genetic Improvement, Huazhong Agricultural University, Wuhan 430070, China; abdullahshalamni@nwafu.edu.cn
* Correspondence: jingxiuqing0615@163.com

Supplemental tables

Table S1. Analysis of physicochemical properties of SiMRLK family proteins.

Name	MV	Ip	Charge	II	AI	GRAVY	Amino Acid Comosition %
SiMRLK1	113016.22	8.08	13.5	39.71	88.88	-0.196	Leu (11.6), Ser (9.9), Gly (7.9), Asn (6.7)
SiMRLK2	102653.69	7.98	12.5	40.28	94.43	-0.185	Leu (12.1), Ser (9.2), Gly (7.3), Ile (7.3)
SiMRLK3	96664.58	6.25	2	38.91	87.35	-0.192	Ser (9.9), Leu (9.4), Gly (7.0), Ala (6.8)
SiMRLK4	92489.18	6.8	6.5	34.61	85.83	-0.205	Leu (9.8), Ser (8.4), Gly (8.2), Ala (7.3)
SiMRLK5	118,423.60	8.12	12	39.51	92.2	-0.115	Leu (12.3), Ser (10.0), Gly (8.5), Ala (6.7)
SiMRLK6	113016.08	6.89	5.5	38.14	87.82	-0.132	Leu (12.0), Ser (10.4), Gly (8.4), Thr (6.9)
SiMRLK7	108841.77	8.48	17.5	35.64	92.83	-0.053	Leu (12.5), Ser (10.5), Gly (7.6), Ala (6.7)
SiMRLK8	113024.98	8.48	17.5	39.07	93.12	-0.064	Leu (12.8), Ser (9.0), Ala (7.8), Gly (7.1)
SiMRLK9	97,435.12	5.55	-9	37.81	82.37	-0.162	Leu (9.6), Ala (8.5), Ser (8.5), Gly (7.7)
SiMRLK10	92175.01	6.87	5	26.17	89.92	-0.105	Leu (10.5), Ser (9.2), Val (8.2), Gly (7.7)
SiMRLK11	95,058.23	6.15	1	33.47	80.13	-0.21	Ser (9.6), Leu (9.2), Thr (8.1), Gly (8.0)
SiMRLK12	94,978.74	5.53	-10	36.59	81.16	-0.174	Ala (9.6), Leu (8.2), Ser (7.9), Val (7.8)
SiMRLK13	93,155.95	6.27	1	41.87	88.48	-0.129	Leu (11.1), Ala (9.4), Ser (9.3), Val (7.0)
SiMRLK14	92,969.69	5.86	-3.5	36.82	88.49	-0.011	Ala (10.8), Leu (9.9), Ser (9.9), val (9.2)
SiMRLK15	112,625.60	6.47	3.5	35.74	88.88	-0.16	Leu (11.0), Ser (9.7), Gly (8.2), Asn (7.0)
SiMRLK16	97902.22	6.12	-0.5	33.00	91.12	-0.097	Leu (12.6), Ser (11.4), Gly (8.0), Val (6.7)
SiMRLK17	96936.85	5.93	-2	32.83	93.51	-0.105	Leu (12.6), Ser (11.6), Gly (8.2), Asn (6.9)
SiMRLK18	111735.65	7.11	5.5	30.73	90.42	-0.12	Leu (12.6), Ser (10.7), Gly (8.0), Asn (7.4)
SiMRLK19	92167.94	6.34	2.5	37.69	90.13	-0.137	Leu (10.4), Ser (10.0), Val (7.2), Thr (6.8)
SiMRLK20	92,724.08	7.92	8	35.65	90.14	-0.07	Leu (10.2), Ser (10.1), Ala (8.2), Ile (6.5)
SiMRLK21	95,724.24	5.77	-5	32.07	79.88	-0.173	Ser (9.3), Leu (8.9), Gly (8.9), Ala (8.1)
SiMRLK22	92484.17	9.06	18.00	43.18	84.38	-0.098	Ala (11.6), Leu (9.5), Ser (9.3), Gly (8.7)
SiMRLK23	93597.62	5.95	-2	44.3	82.83	-0.115	Ala (10.1), Ser (9.4), Leu (9.2), Val (7.9)

List of abbreviation: MV: molecular weight; pI: isoelectric point; II, Instability index; AI, Aliphatic index; GRAVY: Grand average of hydropathicity, GRAVY.

Table S2. The conserved motif analysis of the SiMRLK family proteins in foxtail millet.

Motifs No.	sequences	E-value	Sites	Width	Logo
1	WKTRLEICJGAARGLHYLHEGSSIRIIHRDVKASNI LLDEBLNAKVSDFG	3.3e-687	23	50	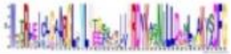
2	QGKREFLTEIEMJSRLRHRNLVSLIGCCDEGNEMJ LVYEYMENGSLRRHL	3.8e-659	23	50	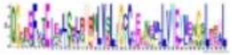
3	KTHVSTAVAGTFGYLDPEYARRGQLTEKSDVYSF GVVLLEVLCGRPNIBP	4.4e-646	22	50	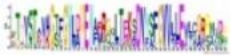
4	ARMSPSSLRYYGJGLENGNYTVKLQFAEIAFTDDQ TWQSLGKRVFDIYIQ	5.1e-252	10	50	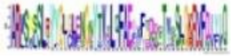
5	JGEGGFGKVYKGVLEDGTVVAVK	3.6e-246	23	23	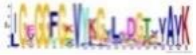
6	VDPRJGGEYSPEELLKFAEVALKCTADSPVQRPSM SDVVWMLEGALZLQE	8.5e-374	23	50	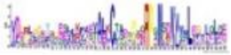
7	AVTKTYTAPVTKNFLEIHLFWAGKGTCCIPTQGY YGPLISAJSVTPNFTP	3.4e-239	10	50	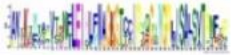
8	ELGNLSKLEQLYJSSSGLSGPLPSTFSKLTNLKILRA SDNDFSGKIPDFJ	4.3e-175	13	50	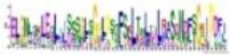
9	CLQRDTPCFLGSPQYSSFAVDCGSNRSMSGSDNSM YDPDPADLGPASYYV	2.2e-126	6	50	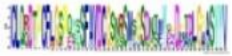
10	GPIPTSLSNLTNLTSLRIGDJVGGSSSLPFJSNMTSL KTLVLRNCRISGN	4.2e-136	9	50	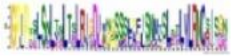

Table S3. Primer sequences for qRT-PCR used in the study.

Gene Name	Gene_Symbol	Primer Sequences
SETIT_016192mg	qSiMRLK1-F	GTGAGGCTCTTTGGTTGTTG
	qSiMRLK1-R	TTCAACTGATGTTCTGAGCGA
SETIT_016287mg	qSiMRLK3-F	ACTGTACCTATCCTCGAGCTG
	qSiMRLK3-R	CATTGCTGGAGAGATCCAGGT
SETIT_028810mg	qSiMRLK7-F	GGCCCGGATGAAGAAGTTAC
	qSiMRLK7-R	ACTGATAATATTAGGTTAGTCAGGC
SETIT_021180mg	qSiMRLK11-F	TCCCGTTTTCGTGCTTCGTA
	qSiMRLK11-R	ACTCCAGGTGACACAGCAAG
SETIT_009354mg	qSiMRLK19-F	AAAGCTTCGGTTTTCCGTGC
	qSiMRLK19-R	CGTCGGTGGCGGAATTTTAC
SETIT_034221mg	qSiMRLK23-F	AACTACCTCGTCCGTCTCCA
	qSiMRLK23-R	AGTCCAGGTAAAAGGCCTGC
SETIT_026509mg	qSiActin-F	CGCATATGTGGCTCTTGACT
	qSiActin-R	GGGCACCTAAATCTCTCGC
